# CCDB: A database for exploring inter-chemical correlations in metabolomics and exposomics datasets

**DOI:** 10.1016/j.envint.2022.107240

**Published:** 2022-04-18

**Authors:** Dinesh Kumar Barupal, Priyanka Mahajan, Sadjad Fakouri-Baygi, Robert O. Wright, Manish Arora, Susan L. Teitelbaum

**Affiliations:** Department of Environmental Medicine and Public Health, Institute for Exposomic Research, Icahn School of Medicine at Mount Sinai, 17 E 102nd St, CAM Building, New York 10029, USA

**Keywords:** Metabolomics, Inter-chemical correlation, Exposomics, Biomonitoring, Metabolic pathways, NHANES, Database, Software

## Abstract

Inter-chemical correlations in metabolomics and exposomics datasets provide valuable information for studying relationships among chemicals reported for human specimens. With an increase in the number of compounds for these datasets, a network graph analysis and visualization of the correlation structure is difficult to interpret. We have developed the Chemical Correlation Database (CCDB), as a systematic catalogue of inter-chemical correlation in publicly available metabolomics and exposomics studies. The database has been provided via an online interface to create single compound-centric views. We have demonstrated various applications of the database to explore: 1) the chemicals from a chemical class such as Per- and Polyfluoroalkyl Substances (PFAS), polycyclic aromatic hydrocarbons (PAHs), polychlorinated biphenyls (PCBs), phthalates and tobacco smoke related metabolites; 2) xenobiotic metabolites such as caffeine and acetaminophen; 3) endogenous metabolites (acyl-carnitines); and 4) unannotated peaks for PFAS. The database has a rich collection of 35 human studies, including the National Health and Nutrition Examination Survey (NHANES) and high-quality untargeted metabolomics datasets. CCDB is supported by a simple, interactive and user-friendly web-interface to retrieve and visualize the inter-chemical correlation data. The CCDB has the potential to be a key computational resource in metabolomics and exposomics facilitating the expansion of our understanding about biological and chemical relationships among metabolites and chemical exposures in the human body. The database is available at www.ccdb.idsl.me site.

## 1. Introduction

Combined exposures to millions of different chemicals and its impact on the health and development of human body is a major component of the exposome (Vermeulen et al., 2020). The chemical exposome is made up of nutrients and environmental non-food chemicals, consisting of natural and synthetic exogenous compounds (Barupal and Fiehn, 2019; Matta et al., 2020; Rappaport et al., 2014). After entering the body, through biotransformation they also become part of the metabolome, which includes metabolic end products of the host and its commensal microbiota. This chemical space (e.g. industrial chemicals, nutrients, drugs, and bioactive internal molecules such as hormones and oxylipins) has significant influence on health trajectories and chronic health outcomes and is implicated in all diseases, including cancer as well as neurological, cardiovascular, and respiratory diseases (Drouin-Chartier et al., 2021; Jobard et al., 2021; Loftfield et al., 2021; Needham et al., 2021; Nemet et al., 2020; Nymand Ennis et al., 2019; Peters et al., 2021; Petrick et al., 2020; Schillemans et al., 2021; Tahir et al., 2021; Vangipurapu et al., 2020). Emerging evidence demonstrates that the scale, magnitude, and structural diversity (Guha et al., 2016; Rappaport et al., 2014) of the internal chemical space is vast and that many chemicals could be classified together because they are structurally and functionally related to each other (Paul-Friedman et al., 2019; Richard et al., 2021; Zimmermann et al., 2019). A systematic understanding and cataloging of targeted and untargeted analyses of small molecules measured in biospecimens is needed, as such datasets are critical to translate the information gathered from exposomics and metabolomics projects (Hendrix et al., 2015). These key datasets include: 1) population-scale biomonitoring surveys; 2) targeted analysis of multiple analytes in hypothesis-driven studies (typically 10–100); and 3) untargeted analysis of thousands of chemicals using a high-resolution mass spectrometry instrument (Barupal et al., 2021a; David et al., 2021). They cover key high priority exposome chemicals (Barupal et al., 2021b) including carcinogens (Hecht et al., 2016; Park et al., 2021), endocrine disrupters (Kassotis et al., 2020) and industry chemicals (Shearer et al., 2021). These core datasets support different statistical and bioinformatics analyses to reveal novel risk factors, hidden metabolic pathways, detrimental exposures and biomarkers for disease.

**Fig. 1.**
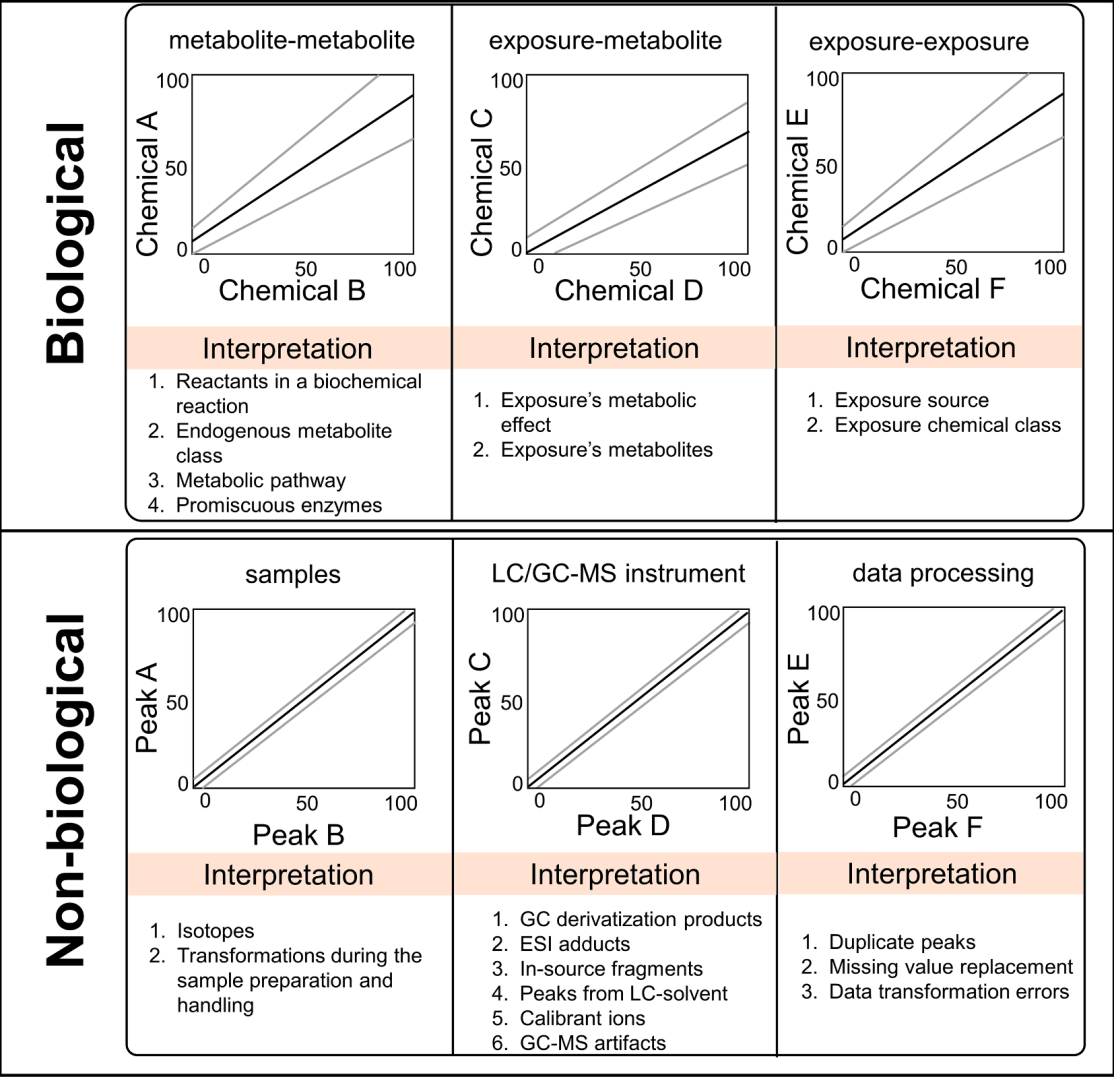
Probable interpretations of correlation in targeted and untargeted GC/LC-HRMS datasets.

Computing the correlation coefficient using intensities of two chemicals is a fundamental statistical approach classically used to study enzyme kinetics (Frieden et al., 1976) and biotransformation (Hoffman et al., 1990). For modern multi-analyte targeted and untargeted assays, a pair-wise correlation matrix among detected chemicals is computed for almost every study because this matrix can be used to assess chemical clustering (Barupal et al., 2019a), peak annotation (DeFelice et al., 2017), heatmaps (Shen et al., 2020), and correlation network visualization (Barupal et al., 2019a). Correlation among gene expression data is often interpreted as evidence of a co-regulatory pathway such as a common transcription factor that controls expression of a group of genes (Obayashi et al., 2019; Yin et al., 2021). As a corollary, with chemicals, correlation can reflect common exposure origins (Edmands et al., 2015) as well as chemical disposition, such as absorption pathways, biotransformation (Frederiksen et al., 2010; Saravanabhavan et al., 2013) and elimination as seen in drugs and their metabolic products (Guo et al., 2020; Guthrie et al., 2019). For exposomic projects, the probable interpretation of inter-chemical correlations is summarized in Fig. 1. The biological interpretation covers both kinetics (i.e. the metabolic fate of a chemical (Cohen et al., 2018)) and dynamics (i.e. the toxic effect of chemical exposure). The system connects to key metabolic pathways (Chen et al., 2020), and creates logical groupings of similar exposures in a chemical class (Barupal et al., 2019a). It can also indicate that two chemicals share an exposure source, such as occupation, consumer products (Stanfield et al., 2021), or food (McKillop et al., 2021). Despite the utility and application of inter-chemical correlation data, a database of these inter-chemical correlations has not yet been developed.

Metabolomic correlation network analyses show that chemically similar compounds and compounds belonging to the same pathway tend to show a higher correlation coefficient (Li et al., 2017; Liang et al., 2020; Toledo et al., 2017). However, creating and analyzing those networks for large and comprehensive metabolomics datasets that often have over ten thousand reported peaks is computationally challenging. It is even more difficult to create and analyze such network graphs for metabolomics datasets that are generated using multiple LC/GC assays (e.g. reverse phase (RP) and hydrophilic interaction liquid chromatography (HILIC) modes) for hundreds of samples (Barupal et al., 2019b). There is a need to catalogue these correlations in a systematic database for mining them in various interpretational contexts.

Herein, we describe a new database, CCDB, which catalogues pairwise inter-chemical correlations from publicly available metabolomics and exposomics studies. It is the largest database of pairwise correlations to date and provides new opportunities for interpreting metabolomics datasets for structural and biological relationships. The database is publicly available at www.ccdb.idsl.me.

## 2. Methods

### 2.1. Selection of studies

Table 1 provides the list of 35 studies and the details about the number of compounds and samples. For the development of the database, we constrained our approach to human specimen studies having at least 50 samples. To include a study in the CCDB, the data were reformatted into CCDB Excel template (SI File 1). The template requires three sheets 1) “data_dictionary” which contains the metadata for annotated and unannotated compounds 2) “data_matrix” which contains the intensity data for all peaks and 3) “sample_metadata” which contains the information about each sample. If data from different chromatography and ionization modes were available, data were stacked in the “data_dictionary” and “data_matrix” sheets. If data were not scaled or normalized, we applied a log2 transformation before computing the correlation.

### 2.2. Processing of untargeted metabolomics studies

Only untargeted liquid chromatography high resolution mass spectrometry studies were selected. For each selected untargeted study (Table 1), we searched for a set of data types in the EBI-MetaboLights and Metabolomics WorkBench repositories. The set included 1) intensity values for annotated peaks 2) intensity values for un-annotated peaks 3) sample metadata and 4) metadata for the annotated peaks. For each reported peak, information about the analysis mode (reverse phase or hydrophilic interaction liquid chromatography) mass to charge ratio and retention time were collected in the “data_dictionary” tab in the CCDB template (https://github.com/idslme/chemcordb/blob/main/MTBSL204_INPUT.xlsx).

### 2.3. Processing of the National health and Nutrition Examination Survey (NHANES) data

Laboratory data for continuous variables were downloaded from the NHANES website (https://wwwn.cdc.gov/nchs/nhanes/search/datapage.aspx?Component=Laboratory) in the SAS export format (.XPT). Variables that reflected a chemical entity were used for calculating the inter-chemical correlation data (Table S1). Data files were imported in the R programming language and merged using the NHANES SEQN number as the linking identifier. NHANES data were used for computing correlation statistics without any transformation, normalization and scaling. Survey design weights do not affect the inter-chemical correlations, so they were not taken into account.

### 2.4. Processing of datasets generated by Metabolon Inc. platform

Metabolomics datasets generated by the Metabolon Inc. company available in the supplementary section of a published article (Germain et al., 2020) or via metabolomics repositories were included in CCDB. The company provides datasets with up to 2,000 high-confidence chemicals reported for blood and urine specimens. If these data were not scaled or normalized, we applied a log2 transformation before computing the correlation. For the CCDB input format, only metabolite names reported in the table were used in the “data dictionary” tab of the CCDB format.

### 2.5. Correlation calculation

The Pearson correlation coefficient was used for computing a pairwise correlation among reported peaks within each study using the cor function available in the WGCNA R package (Langfelder and Horvath, 2008). A correlation between two intensity vectors was computed only if they had at least 10% non-zero values. We did not compute any p-values for the correlation statistics given that our goal was to create a database of inter-chemical correlations, not to find a biomarker of phenotype. Therefore, the application of a false discovery rate correction was not required. If p-values were computed, they would be expected to be extremely small considering the large sample sizes of the selected studies.

Overall average detection rate across all studies were 60% of above (Table S2). However, it is common for human biospecimen studies that several compounds, especially exposure-related are detected only in a fraction of samples in a study. For example, Fluoro-phenoxybenzoic acid was found only in 170/2694 (6.3%) samples (https://wwwn.cdc.gov/Nchs/Nhanes/2007-2008/UPHOPM_E.htm#URD4FPLC). Therefore, for NHANES we have used a criteria that a compound must be detected in at least 100 samples to be included in the computation of inter-chemical correlations.

### 2.6. CCDB indexing

For each selected study, a unique name directory was created in a webserver’s filesystem and the pairwise correlation data were saved inside the corresponding directory. For each compound, a vector of correlation against all other chemicals in the study were computed and then stored in the file system. For the naming convention, a distinct study-specific identifier was assigned to each reported chemical. Linux operating system Ubuntu 20.04 was used for the webserver.

### 2.7. Online interface and querying the CCDB

The online front interface was developed using the AngularJS 1.5 javascript framework and bootstrap. On the backend, a nginx proxy server was used to route the web requests to the data indexed in the CCDB. The opencpu framework (https://www.opencpu.org/) in R was used as a middleware to process each web request. For biomonitoring (NHANES), Metabolon Inc’s datasets and untargeted full-scan datasets, three separate types of web-interfaces were developed. For visualizing the correlation data online, Vis.JS javascript library was utilized. If there are more than 100 hits that pass the correlation threshold only the first hundred hits are visualized in the compound centric network and full data were provided as Cytoscape network file.

For each study, a specific web-address was created (Table S3). For NHANES data, the query parameter is a variable identifier provided in the Table S1. For Metabolon Inc’s datasets, chemical names were utilized. For full-scan untargeted datasets, *m*/*z* with a mass tolerance was used to retrieve the matched peaks in the database. To obtain putative annotation hits, *m*/*z* values were matched against a list of compounds that have been associated with a published paper.

### 2.8. Chemical similarity enrichment (ChemRICH) analysis

ChemRICH is a database independent and *p*-value distribution-based approach to rank the chemical sets that are associated with an exposure (Barupal and Fiehn, 2017). As an example, cor.test function in R was used to obtain *p*-values and estimates for the correlation between Perfluorooctanoic acid (PFOA) intensities and other chemicals from the study IDSLCCDB0001 (Needham et al., 2021). These results and the subpathway information made available by the Metabolon Inc’s report were used as an input for the chemical similarity enrichment analysis using the ChemRICH software (Barupal and Fiehn, 2017).

### 2.9. Data and code availability

All data and resources are available at www.ccdb.idsl.me site. Core scripts to compute the inter-chemical correlation data from biomonitoring and metabolomics studies have been provided at https://github.com/idslme/CCDB.

## 3. Results

### 3.1. CCDB is a comprehensive database of inter-chemical correlations for human biospecimens

To build a comprehensive database of inter-chemical correlations in human biospecimens, we found three types of chemical analyses that should be covered. These included 1) biomonitoring surveys that have used a targeted analysis for chemical panels 2) metabolomics datasets having structurally annotated peaks 3) untargeted LC/GC-HRMS datasets having primarily unannotated peaks. In the first version of the CCDB, 35 studies were included (Table 1). The coverage for specimen types was 28 (blood), 3 (urine), 4 (stool). The number of individual participants was 107,258 for NHANES with 607 laboratory measurement variables. For 18 datasets that were generated by Metabolon Inc, the sample size ranged from 52 to 1,336 with the reported peak count ranging between 517 and 1989. For 16 full-scan untargeted LC-HRMS studies, the sample size ranged between 51 and 781 with a reported peak count of 459 to 81867, and 8 studies had reported only unannotated peaks that were referenced using *m*/*z* and retention time values. To update the database, we plan to regularly screen publicly available datasets in the Metabolomics Workbench, EBI MetaboLights, GNPS-Massive and consortium/cohort specific repositories and supplementary tables for published papers and include the relevant studies in the CCDB database. By covering three types of chemical measurement datasets, CCDB can provide unique opportunities to not only learn about the biological relationships among metabolites, but also prioritize chemicals that are yet to be annotated in untargeted LC/HRMS datasets.

### 3.2. A large number of inter-chemical correlations were observed in the catalogued studies

**Fig. 2.**
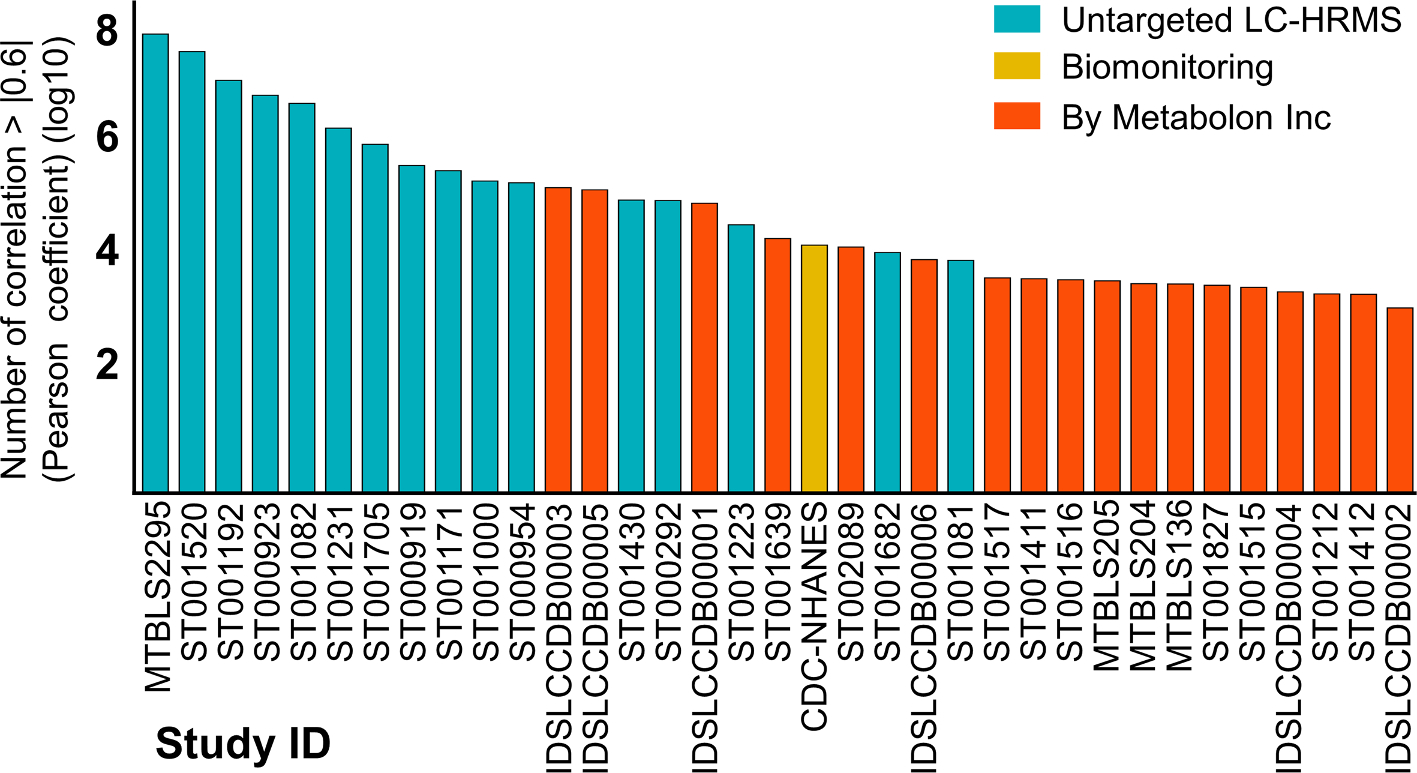
Prevalence of strong inter-chemical correlations across 35 studies in the CCDB. These are unique correlations. See the Table 1 for the description of each study and number of compounds. Table S3 shows the chemical detection rate across the indexed studies.

To populate the database, pair-wise correlations among reported chemicals were computed for each selected study. A computational pipeline has been established for an efficient indexing of a new dataset in the database. For that, a minimal level of manual curation was needed to prepare the dataset in the required format (See methods). We investigated the prevalence of strong inter-chemical correlations across the catalogued studies. A total of 121.4 million inter-chemical correlations across the studies passed a threshold of 0.6 Pearson coefficient, indicating the large-scale and magnitude of strong correlation patterns that exists among chemical compounds measured for human biospecimens (Fig. 2). More of these correlations were observed for untargeted datasets which had thousands of mostly unannotated peaks. We noticed that endogenous compounds tend to show a higher number of significant correlations in comparison to exogenous and xenobiotic compounds (Fig. S1). This suggested that at a lower correlation threshold level, we can capture new relationships among chemicals that would otherwise be missed if the correlation data is visualized as a network graph created using a stringent threshold.

For example, by a Pearson coefficient cutoff of 0.4, we have noticed a relationship among blood glucose and acyl-choline lipids (Fig. S2) in the study MTBL136 (Stevens et al., 2018) which will be missed on a cutoff of 0.6. This association has been linked with energy disturbance and implicated in diabetes and chronic fatigue disease related studies. This underscores the need to access the correlation data in a flexible and interactive approach so we can capture both the known and novel types of functional and biological relationships among reported chemicals.

### 3.3. Dataset type specific web-interfaces provided access to correlation data for both annotated and unannotated compounds

Because a large number of inter-chemical correlations were observed in the selected studies, it was not practical to visualize them all as a global network in Cytoscape network visualization (Shannon et al., 2003) or any other network graph visualization software unless the network graph is created using very stringent correlation thresholds, which will likely miss biological insights. Therefore, we stored all the correlation data for each compound from each study in a web-server’s file system. This allows us to readily load the correlation vector in the computer memory without the need to re-calculate them and enabled a faster response time for the online visualization. A network-based visualization highlighted a compound centric view of inter-chemical correlations, which can be updated by different correlation thresholds. A compound centric view was found to be a cleaner, readable and meaningful visualization than creating a network graph of all compounds reported in a study. It enables a focused investigation of a single compound and its chemical and biochemical relationships with other chemicals in a study. Three types of web interfaces were developed to provide a tailored access inter-chemical correlation data for biomonitoring, annotated peaks and unannotated data in metabolomics and exposomics assays (Fig. S3–5). These interfaces enabled queries by chemical names, CAS numbers, NHANES identifiers and mass to charge (*m*/*z*) ratio. For untargeted assays, data from different analysis modes were stacked which allowed to find peaks from the same compound in two analysis modes such as an ESI positive and negative or HILIC (+) or RP (+) (Fig. S6). Network data were also provided as Cytoscape network files to enable additional visualization strategies. These simple and flexible web-interfaces allowed a seamless and interactive access to the inter-chemical correlation data for a chemical from a study.

### 3.4. Compounds from a chemical class correlated strongly with each other in the NHANES biomonitoring dataset

**Fig. 3.**
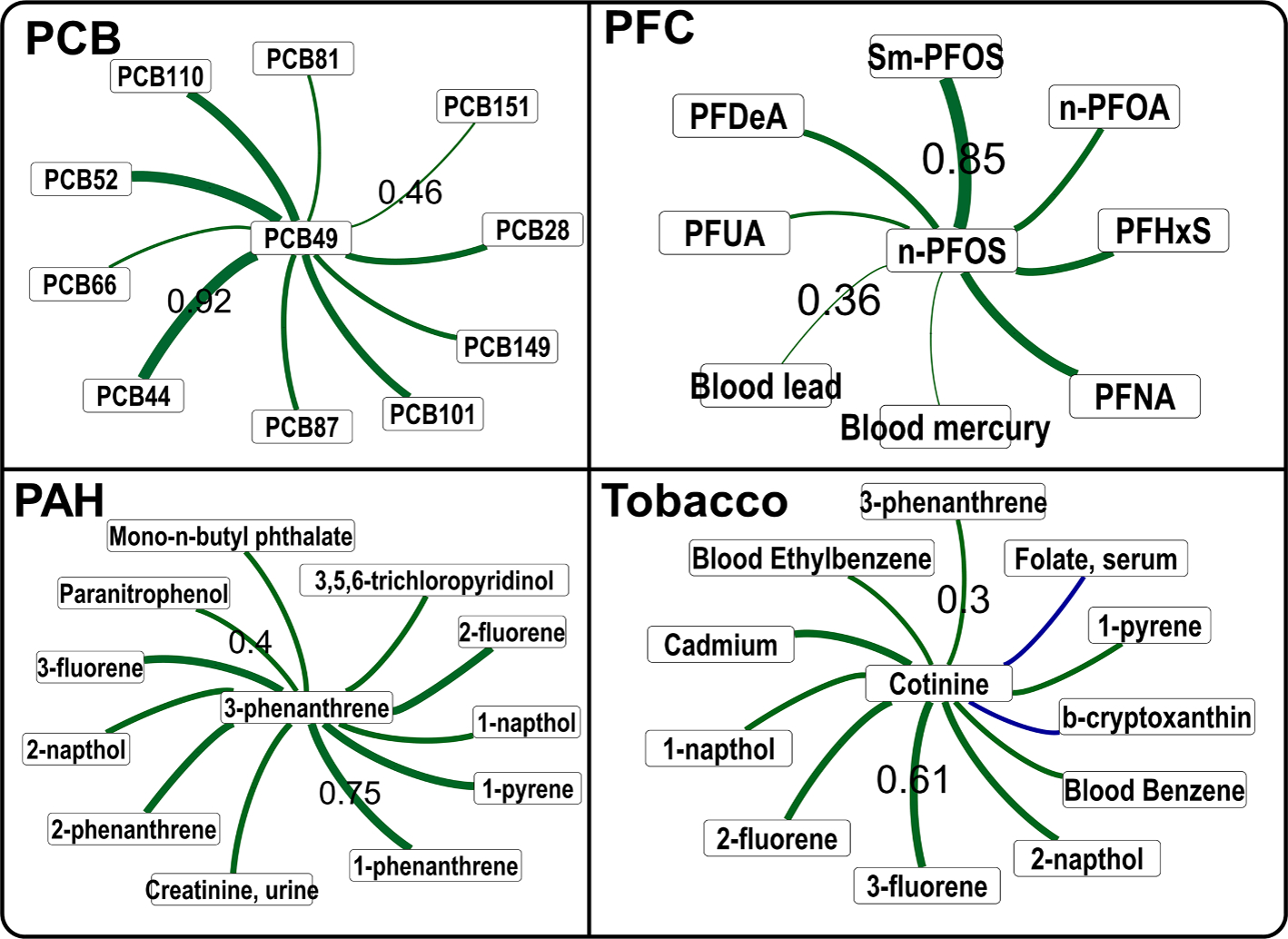
Correlations among chemicals within a class or having same source origin in the NHANES dataset. The correlation cutoff was 0.3 for PCB, PFC and Tobacco compounds, and 0.4 for PAHs. Online network can be accessed at the site - https://chemcor.idsl.site/originaldata/biomonitoring/#?studyid=NHANES. Edge thickness shows the correlation strength, by only the minimum and maximum correlation values are labelled on the edges for clarity. Thickness of edges are not comparable in two network figures. Abbreviations: Perfluorodecanoic acid (PFDeA), Perfluorohexane sulfonic acid (PFHxS), Perfluorononanoic acid (PFNA), Perfluoroundecanoic acid (PFUA), n-perfluorooctanoic acid (n-PFOA), n-perfluorooctane sulfonic acid (n-PFOS), Perfluoromethylheptane sulfonic acid isomers (SmPFOS), Polychlorinated Biphenyls (PCB); polyaromatic hydrocarbons (PAH), Perfluorinated compounds (PFC).

First, we asked if compounds from a known chemical class correlate with each other and can be retrieved by querying a single chemical. We have observed that chemicals from well-recognized environmental exposures PCB, PFC and PAH groups indeed correlated with a representative chemical from these classes (Fig. 3). This probably suggested a common source of exposure for these chemicals. When cotinine, a biomarker of tobacco smoke was queried, it retrieved many other tobacco smoke related chemicals, providing a quick overview of biomarkers of smoke exposures.

This compound-centric retrieval of inter-chemical correlations in the NHANES biomonitoring dataset suggested that chemical exposures with similar structure and origin correlates strongly with each other.

### 3.5. Stronger correlations among compounds belonging to a chemical class in metabolomics datasets

**Fig. 4.**
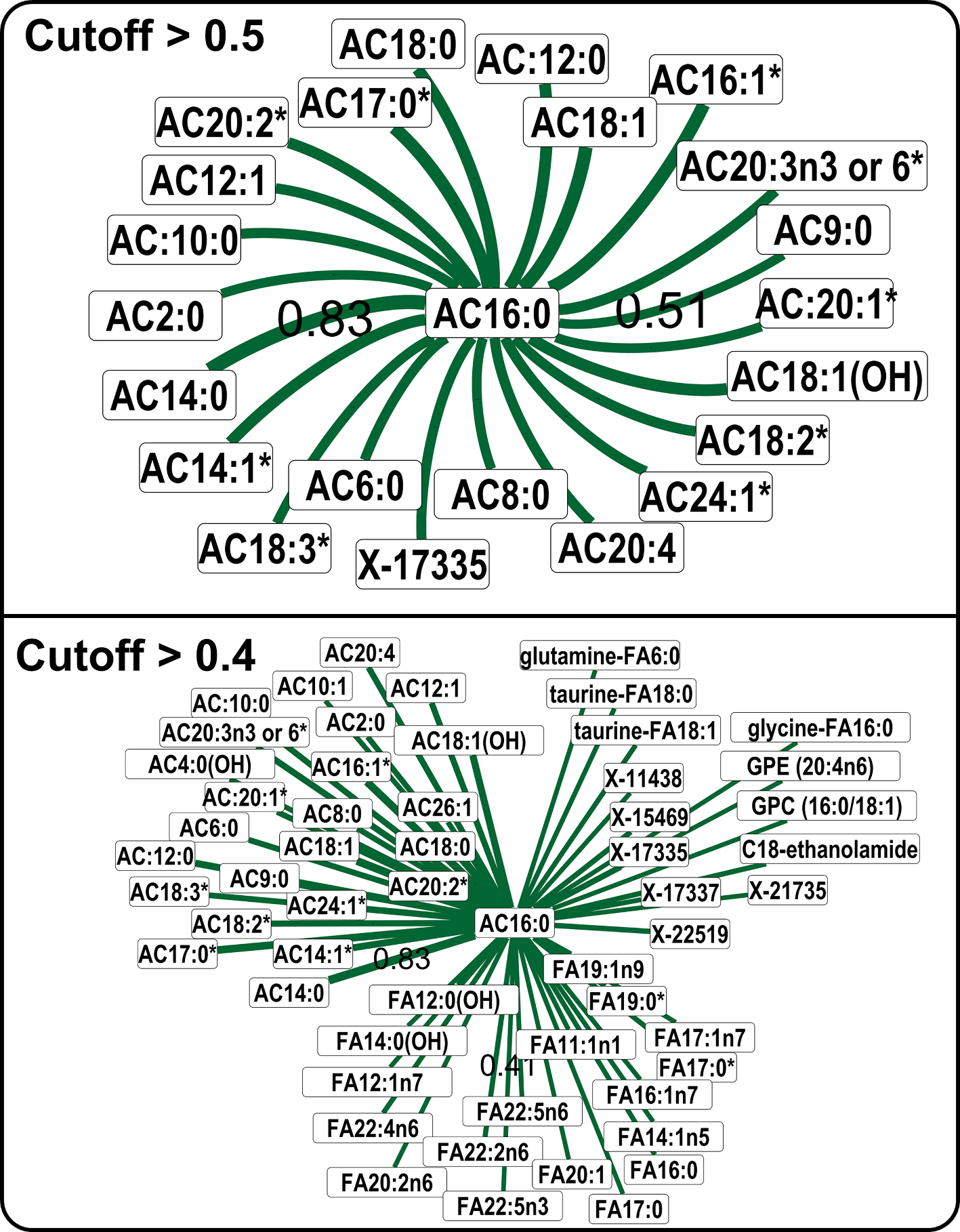
Compounds correlation with acylcarnitine 16:0 in the study ST002089. Edge thickness shows the correlation strength, by only the minimum and maximum correlation values are labelled on the edges for clarity. Thickness of edges are not comparable in two network figures. Abbreviations: acyl-carnitines (AC). Fatty acid (FA), glycerophosphoethanolamine (GPE), glycerophosphocholine (GPC).

Next, we investigated if endogenous metabolites from a chemical class correlated with each other in a metabolomics dataset. We queried a ubiquitous endogenous blood metabolite, C-16 carnitine and retrieved its neighbors in the ST002089 study. At the Pearson correlation cutoff of 0.5, we retrieve mostly other saturated and unsaturated carnitines (Fig. 4). However, at the 0.4 Pearson correlation cutoff, we found that carnitines have biochemical relationships with fatty acids and acylcarnitines.

We learned that structurally similar compounds from an endogenous chemical class can have a high correlation coefficient among them, suggesting an enzyme activity that can react on any member of a chemical class, for instance the carnitine palmitoyltransferase I enzyme. As the Pearson correlation cutoff was lowered, we found long-distance biochemical relationships suggesting different chemical classes that may belong to a metabolic pathway, for instance, fatty acids and acylcarnitines. It also highlighted the unidentified metabolites that correlated strongly with C16-carnitine in the Metabolon Inc’s report may belong to the acyl-carnitine chemical class. In summary, by modifying the correlation cutoff, the CCDB interface enables retrieval of short and long-distance biochemical relationships in a metabolic network around a single chemical. This can be used for hypothesizing novel biochemical relationships in untargeted metabolomics datasets.

### 3.6. Products of xenobiotic metabolism

**Fig. 5.**
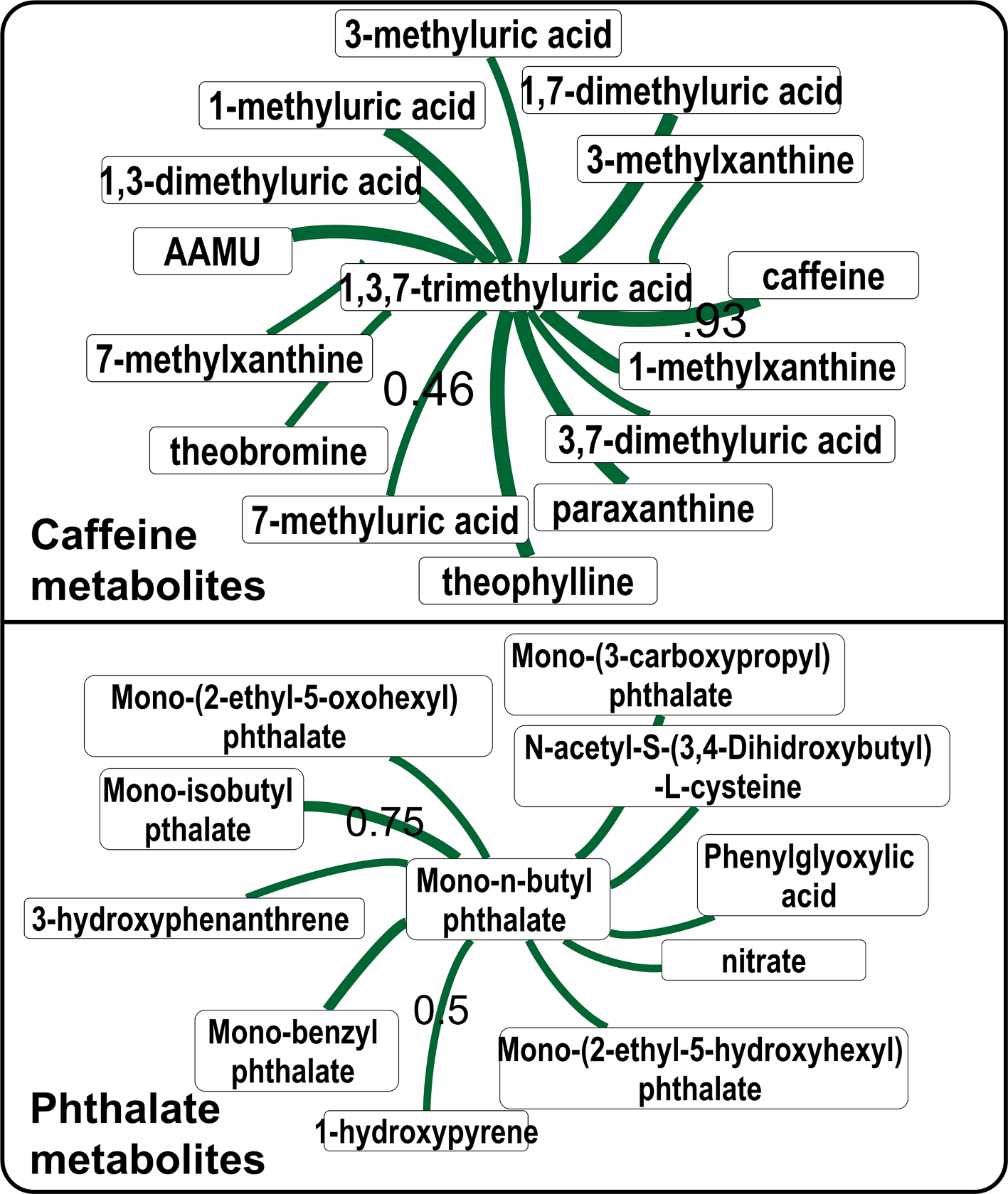
Caffeine and phthalate metabolites in the NHANES survey data. Variable id URXMBP_PHTHTE_D (year 2005–2006) was used for mono-n-butyl phthalate (MnBP). Variable id URXMX7_CAFE_H (year 2013–2014) was used for caffeine. Label on the edges show the Pearson coefficient. Edge thickness shows the correlation strength, by only the minimum and maximum correlation values are labelled on the edges for clarity. Thickness of edges are not comparable in two network figures. Abbreviations: acetylamino-6-formylamino-3-methyluracil(AAMU).

Next, we checked if metabolites of a xenobiotic compound correlate with the parent compound’s levels. In the NHANES biomonitoring survey, several metabolites of caffeine strongly correlated with caffeine levels (Fig. 5, upper panel). The same pattern was found in a metabolomics dataset (Fig. S7). Similarly, metabolites of mono-n-butyl phthalate (MnBP), a commonly used plasticizer correlated with structurally and metabolically related chemicals. MnBP also correlated with other phthalate molecules (Fig. 5 lower panel), indicating common exposure sources. It was expected that people exposed to dibutyl phthalates will excrete MnBP and mono-isobutyl phthalate in their urine (Qian et al., 2015). For acetaminophen, a commonly used over the counter pain-reliever drug, its sulfate metabolite was found to be correlating with other acetaminophen metabolites (Fig. S8).

### 3.7. Putative annotation of peaks in untargeted data by correlation patterns

**Fig. 6.**
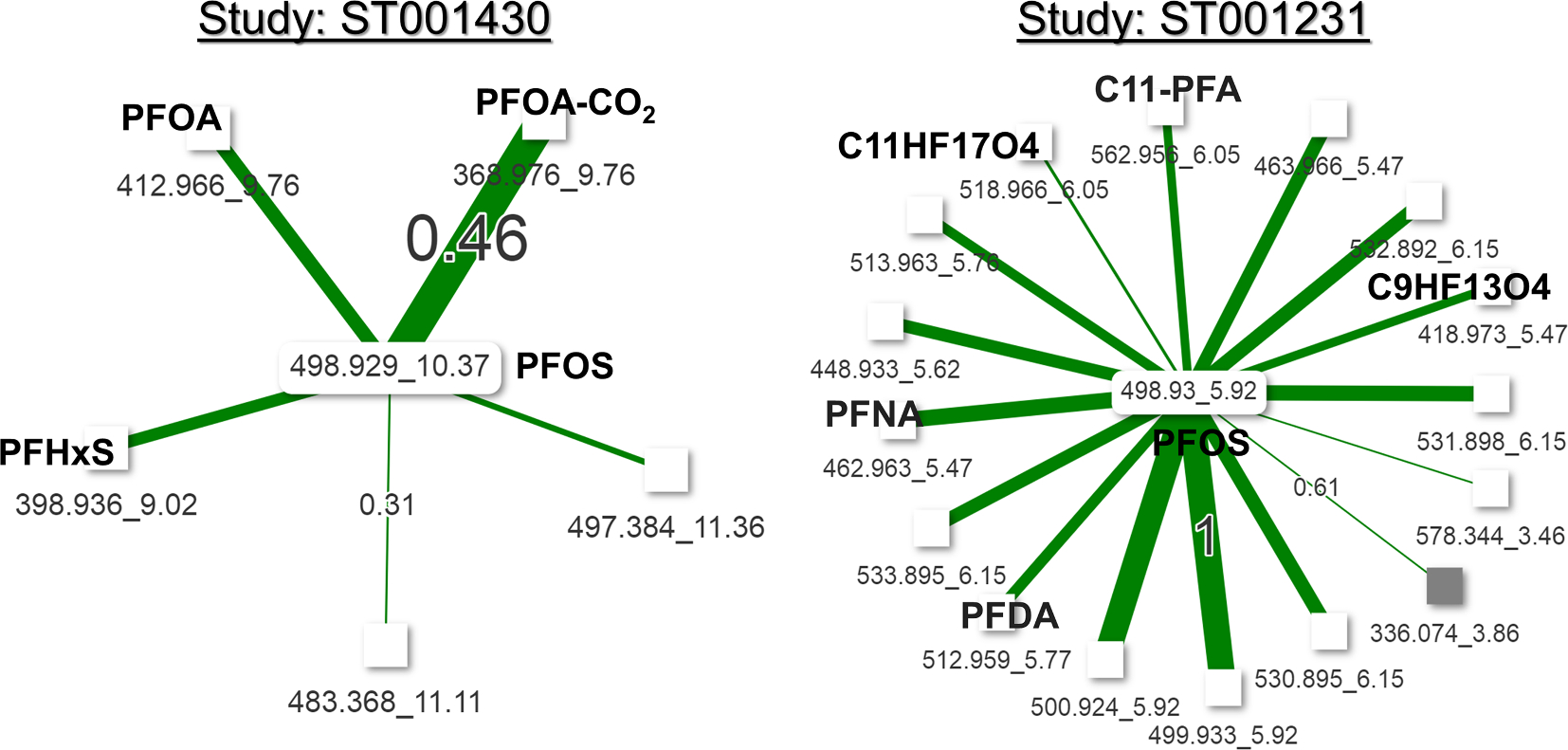
Inter-chemical correlation among PFCs in the untargeted metabolomics datasets. Correlation threshold for ST001430 was 0.3 and for 0.6 for ST001231. White color node mean it was detected in by the reverse phase ESI (−) mode and a grey node means it was detected by a reverse phrase ESI (+) mode. Edge thickness shows the correlation strength, by only the minimum and maximum correlation values are labelled on the edges for clarity. Thickness of edges are not comparable in two network figures.

So far, we have learned from the NHANES and other high quality metabolomics dataset that chemicals within a chemical class or having the same origin or similar pathway tends to show strong correlations. Relying on this information, we explore the untargeted metabolomics datasets to test if *m*/*z* values for chemicals from a chemical class show inter-chemical correlations. To test this, we have queried the *m*/*z* value 498.9291 for the M–H adduct of perfluorooctanesulfonic acid (PFOS) in reverse phase chromatography data for the ST001430 study. It retrieved three other chemicals on in the correlation cutoff of 0.3, which matched to the M–H adducts for other common PFCs - PFOA and PFHxS (Fig. 6). In another untargeted study ST001231, we found that PFOS correlated with many more PFCs compounds (Fig. 6).

### 3.8. Metabolic effect of a hazardous chemical - PFOA

Finally, we asked if we could utilize the inter-chemical correlation data to understand metabolic effects of a chemical exposure. Perfluorochemicals (PFCs) are concerning chemicals for public health. They are exclusively synthetic and accumulate in human body overtime. The ubiquitous exposures to them have been under high priority investigations since they may have contributed to the etiology of a range of chronic diseases. Endogenous metabolites that correlate with PFCs exposures may reflect the biological response to these hazardous chemicals. In several of Metabolon Inc’s reports, Perfluorooctanoic acid (PFOA) peak was annotated and found to be correlated with many chemicals when we indexed these reports in the CCDB.

**Fig. 7.**
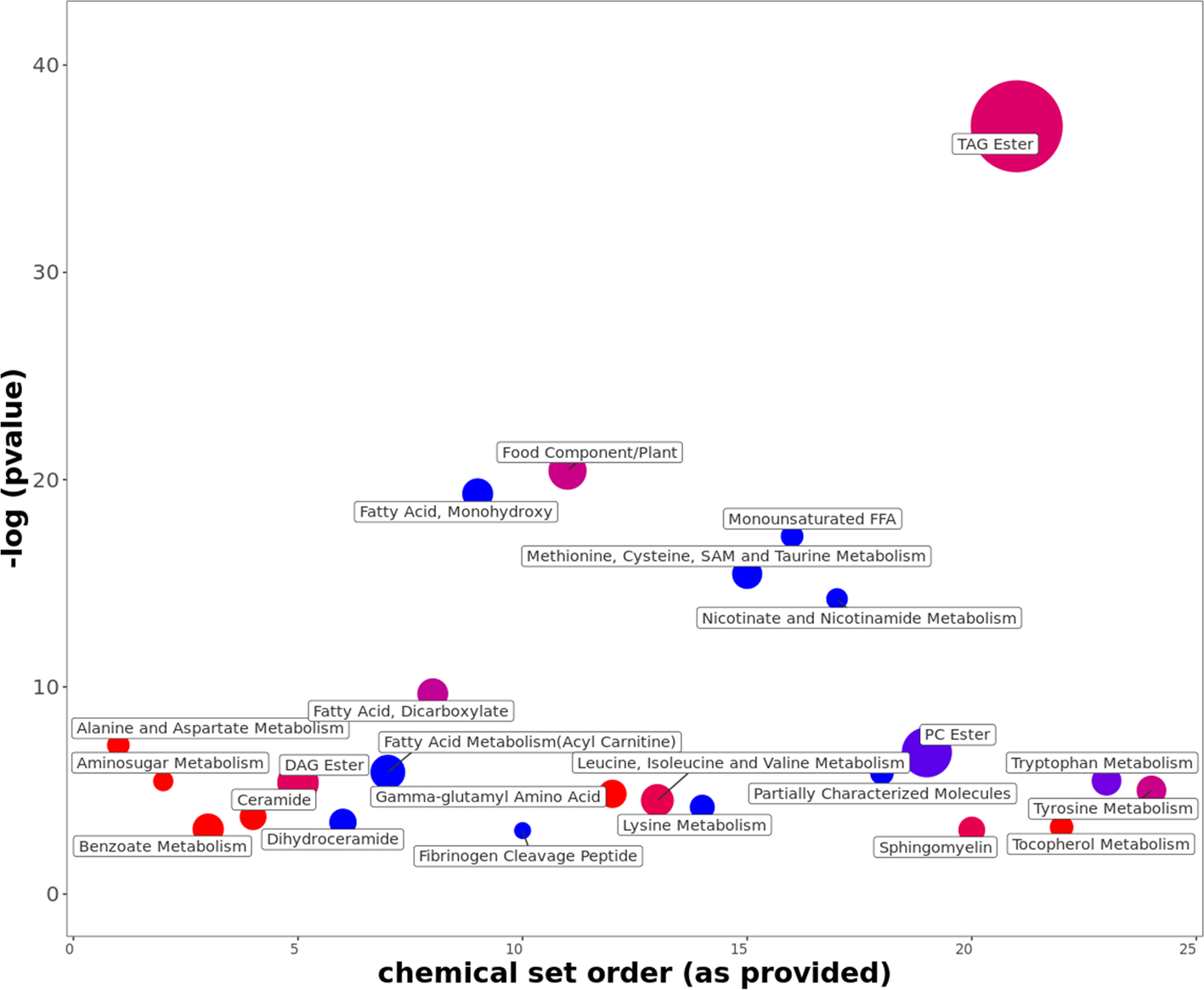
Chemical similarity enrichment analysis of PFOA and its correlation with other metabolites in the IDSLCCDB00001 study.

Many metabolites that correlated with PFOA levels may belonged to the same pathway or chemical class. Identifying these chemical sets can assist in understanding the systematic metabolic effect of PFOA exposure which can span over multiple metabolic pathways (Fig. 7). Therefore, we have utilized ChemRICH analysis (Barupal and Fiehn, 2017) to identify the PFOA associated chemical sets, which suggested that PFOA exposure has a negative effect on most of the lipid sets except triglycerides (Sen et al., 2022; Sinisalu et al., 2020). PFOA exposure may have also induced the amino acid and tocopherol metabolism pathways. This analysis highlighted that CCDB correlation data can also be used for investigating the metabolic hazardous effect of a chemical exposure of public health concern using a chemical set analysis approach.

## 4. Discussion

Inter-chemical correlations in biomonitoring, metabolomics and exposomics datasets is a useful source of information to expand our understanding about the relationships between different metabolites, metabolic pathways and the chemical exposures. There is a need to systematicaly catalogue and preserve these correlation patterns in a database to support useful queries. In this paper, we have presented the CCDB database which aims to build a catalogue of inter-chemical correlation in chemical measurement datasets and then provide users access to the correlation data using a web-interface. As of March 2022, the database includes data from from 35 studies covered. We plan to regularly screen literature as well as metabolomics and exposomics repositories to identify additional studies that can be catalogued in the CCDB. The database currently only hosts studies related to human specimens, however given the generic nature of the catalogued data and indexing pipelines, it will be able to incorporate studies of other species or sources as long as data are provided in the required format. We foresee a regular use of the database in the field of metabolomics and exposomics to explore about the biochemical and chemical relationships around a chemical that has been prioritized by a researcher using statistical or by text mining approaches. We believe the CCDB will be a core database resource in these fields where the interpretation of multi-analyte datasets remains a major challenge.

A large number of significant inter-chemical correlations are ubiquitously observed in these core datasets. An obvious question is “what are the reasons behind these correlations”? At present this is a challenging question because these correlations can be interpreted only in the context of known exposure sources, biochemical absorption pathways and transformation reactions (Barupal et al., 2018). With time, and additional cataloguing of the exposome, the reasons behind these correlations will become more evident. Pathway-centric approaches do not cover many high-priority exposome chemicals, including their chemical classes, source origin and transformation products. The CCDB is designed to address this issue by curating and interpreting inter-chemical correlations in exposomics and metabolomics core datasets while integrating information about functional and structural relationships among chemicals.

In the transcriptomics field, gene correlation or co-expression databases (Lee et al., 2020; Obayashi et al., 2019) have been developed for multiple species and disease conditions. These databases allow the identification of gene function(s) based on the similarity between two gene’s expression levels. They have shown that the similarity in expression levels reflect a shared function or regulation in the genetic networks. CCDB is in line with these databases to provide similar resource for chemicals. For the first time, we developed an inter-chemical correlation database to be used for metabolomics and exposomics hypothesis generation and characterization.

Due to the large number of analytes in targeted and untargeted assays, a traditional correlation network graph of all analytes (Kitagawa et al., 2019; Lau et al., 2018) using Cytoscape (Shannon et al., 2003) or similar software would not be meaningful to explore inter-chemical correlation data because the network graphs would be over-crowded requiring a stringent correlation threshold to draw the edges. Instead, we propose to use a single-compound centric network to generate clear and readable networks that are easy to deploy in online interfaces. We suggest that investigators can explore correlation data in this interactive, compound centric way so that novel relationships among chemicals can be readily explored. In this way, CCDB fullfills critical gaps in the mining of metabolomics correlation data.

CCDB can play a role in peak annotation in untargeted metabolomics, because compounds belonging to the same class, metabolic pathway or source origin tends to correlate with each other. By querying a single compound’s *m*/*z*, we will be able to estimate the chemical class or in some cases the exact identity of a peak, although it will be only based on the MS match against a priority list of chemicals from a database. There is a need to develop further tools to utilize the isotope patterns, MS2 spectra to refine the annotation patterns. For full-scan untargeted datasets, *m*/*z* with a mass tolerance will be used to retrieve matched peaks in the database. In untargeted chemical analyses, many inter-chemical correlations are often observed due to non-biological causes. They are useful in annotating peaks in the untargeted dataset with isotope information (Semente et al., 2021), chemical fragments (J Guo et al., 2021), and errors during data processing, such as duplicate peaks. These annotations can be transferred to other untargeted studies with many unidentified peaks, with the logic that pairs of the same compound will show similar inter-chemical correlation irrespective of analysis platform. It was shown in the example for PFCs and caffeine metabolites (Figs. 7 and S8). It is expected that some of the inter-chemical correlations may not be found across multiple studies or may not have the same strength, which can suggest that the underlying regulatory or source mechanisms are operating differently in two studies. These differences can be considered high priority hypothesis.

In the future version of the CCDB, we may include with more tissue types and clinical outcome datasets (open-access) from the HHEAR program and other NIH supported consortiums. This may enable us to highlight the biomedical relevance of a compound-centric correlation network that is created for a phenotype or outcome. Applications of text mining (Barupal et al., 2021b), chemoinformatics and other bioinformatics resources (Barupal et al., 2018) can also be explored to aid in the interpretation of inter-chemical correlations.

## 5. Conclusions

We describe CCDB, a new key database in the field of metabolomics and exposomics that provides access to fundamental information on the inter-chemical correlations among chemical signals derived from human specimens. The database has a potential to accelerate learning about the chemical and biochemical relationships among reported chemicals. It can be used for prioritizing chemicals, identifying new hypotheses, interpreting metabolomics datasets, annotating peaks in untargeted metabolomics datasets, and for investigating the metabolic effects of a known chemical exposures. Overall, CCDB will start a new wave of database types in the metabolomics and exposomics field that are more interpretive than just a catalogue of information.

## Supplementary Material

1

2

3

FS3

FS2

FS1

FS6

FS4

FS5

FS7

FS8

## Figures and Tables

**Table 1:** Covered studies in the CCDB on March 2022.

Database/Source	Accession ID	Title	Number of Samples	Number of Peaks	Specimen
Metabolomics WorkBench	ST000923	Longitudinal Metabolomics of the Human Microbiome in Inflammatory Bowel Disease (Lloyd-Price et al., 2019)	546	81,867	Stool
Metabolomics WorkBench	ST001000	Gut microbiome structure and metabolic activity in inflammatory bowel disease (Franzosa et al., 2019)	220	8847	Stool
Metabolomics WorkBench	ST001192	A library of human gut bacterial isolates paired with longitudinal multiomics data enables mechanistic microbiome research (Poyet et al., 2019)	180	54,402	Stool
Metabolomics WorkBench	ST001520	Stool unknowns profiled using hybrid nontargeted methods (part-II)(Tanes et al., 2021)	166	54,014	Stool
CDC-NHANES	NHANES	National Health and Nutrition Examination Survey – USA. Continuous NHANES data from 1999 to 2020 period. https://www.cdc.gov/nchs/nhanes/index.htm	107,258 (SEQN IDs)	607 (variables)	Blood/Urine
Metabolomics WorkBench	ST001223	Host Metabolic Response in Early Lyme Disease (Fitzgerald et al., 2020)	518	2193	Blood
Metabolomics WorkBench	ST001081	Combined NMR and MS Analysis of PC patient serum (part-I)(Clendinen et al., 2019)	168	459	Blood
Metabolomics WorkBench	ST001082	Combined NMR and MS Analysis of PC patien serum (part-II)(Clendinen et al., 2019)	265	24,928	Blood
Metabolomics WorkBench	ST001682	Untargeted urine LC-HRMS metabolomics profiling for bladder cancer binary outcome classification	311	982	Urine
EBI MetaboLights	MTBLS136	Serum metabolomic profiles associated with postmenopausal hormone use (Stevens et al., 2018)	1336	1385	Blood
EBI MetaboLights	MTBLS204	Metabolomics analysis of human acute graft-versus-host disease reveals changes in host and microbiota-derived metabolites (Michonneau et al., 2019)	86	801	Blood
EBI MetaboLights	MTBLS205	Metabolomics analysis of human acute graft-versus-host disease reveals changes in host and microbiota-derived metabolites (Michonneau et al., 2019)	112	929	Blood
Metabolomics WorkBench	ST001516	Identification of distinct metabolic perturbations and associated immunomodulatory events during intra-erythrocytic development stage of pediatric Plasmodium falciparum malaria (Abdrabou et al., 2021)	199	668	Blood
Metabolomics WorkBench	ST001517	Identification of distinct metabolic perturbations and associated immunomodulatory events during intra-erythrocytic development stage of pediatric Plasmodium falciparum malaria (Abdrabou et al., 2021)	106	652	Blood
Metabolomics WorkBench	ST001639	Plasma Metabolomic signatures of COPD in a SPIROMICS cohort (Gillenwater et al., 2020)	649	1174	Blood
Metabolomics WorkBench	ST001212	Fish-oil supplementation in pregnancy, child metabolomics and asthma risk (Rago et al., 2019)	577	656	Blood
Metabolomics WorkBench	ST001827	The pregnancy metabolome from a multi-ethnic pregnancy cohort (Colicino et al., 2021)	410	1110	Blood
PMC Open Access	IDSLCCDB00001	Plasma and Fecal Metabolite Profiles in Autism Spectrum Disorder (Needham et al., 2021)	222	1611	Blood
PMC Open Access	IDSLCCDB00002	Potential role of indolelactate and butyrate in multiple sclerosis revealed by integrated microbiome-metabolome analysis (Levi et al., 2021)	180	517	Blood
PMC Open Access	IDSLCCDB00003	Comprehensive Circulatory Metabolomics in ME/CFS Reveals Disrupted Metabolism of Acyl Lipids and Steroids (Germain et al., 2020)	52	1790	Blood
PMC Open Access	IDSLCCDB00004	Plasma Metabolomic Profiling in Patients with Rheumatoid Arthritis Identifies Biochemical Features Indicative of Quantitative Disease Activity (Hur et al., 2021)	128	686	Blood
PMC Open Access	IDSLCCDB00005	Alterations in Polyamine Metabolism in Patients With Lymphangioleiomyomatosis and Tuberous Sclerosis Complex 2-Deficient Cells (Tang et al., 2019)	78	1989	Blood
PMC Open Access	IDSLCCDB00006	Metabolic perturbation associated with COVID-19 disease severity and SARS-CoV-2 replication (Krishnan et al., 2021)	72	1086	Blood
Metabolomics WorkBench	ST002089	Plasma metabolomic signatures of COPD: A metabolomic severity score for airflow obstruction and emphysema (Gillenwater et al., 2021)	1120	1394	Blood
Metabolomics WorkBench	ST001411	Plasma metabolites of lipid metabolism associate with diabetic polyneuropathy in a cohort with screen-tested type 2 diabetes: ADDITION-Denmark (Rumora et al., 2021)	106	991	Blood
Metabolomics WorkBench	ST001412	Metabolomics study in Plasma of Obese Patients with Neuropathy Identifies Potential Metabolomics Signatures (K Guo et al., 2021)	131	842	Blood
Metabolomics WorkBench	ST001515	A Metabolomic Signature of Glucagon Action in Healthy Individuals with Overweight/Obesity Humans (Vega et al., 2021)	187	649	Blood
Metabolomics WorkBench	ST001171	Metabolomics of World Trade Center Exposed New York City Firefighters	248	2504	Blood
Metabolomics WorkBench	ST001430	Metabolic dynamics and prediction of gestational ange and time to delivery in pregnant women (Liang et al., 2020)	781	9651	Blood
Metabolomics WorkBench	ST001705	Machine learning-enabled renal cell carcinoma status prediction using multi-platform urine-based metabolomics (part-I)(Bifarin et al., 2021)	256	7097	Urine
Metabolomics WorkBench	ST000292	LC-MS Based Approaches to Investigate Metabolomic Differences in the Plasma of Young Women after Drinking Cranberry Juice or Apple Juice (Liu et al., 2020)	51	3395	Blood
Metabolomics WorkBench	ST000919	Investigating Eicosanoids Implications on the Blood Pressure Response to Thiazide Diuretics	140	10,322	Blood
Metabolomics WorkBench	ST000954	Explore Metabolites and Pathways Associated Increased Airway Hyperresponsiveness in Asthma	55	7930	Blood
Metabolomics WorkBench	ST001231	Plasma untargeted metabolomics study of pulmonary tuberculosis (Huang et al., 2019)	159	17,146	Blood
EBI MetaboLights	MTBLS2295	High-Precision Automated Workflow for Urinary Untargeted Metabolomic Epidemiology (Meister et al., 2021)	87	655	Urine
