# Supplementary figures and images for: CCDB: A database for exploring inter-chemical correlations in metabolomics and exposomics datasets
Source: Environ Int. Author manuscript; Available in PMC 2022 Jun 14. (PMC9195052; doi:10.1016/j.envint.2022.107240)

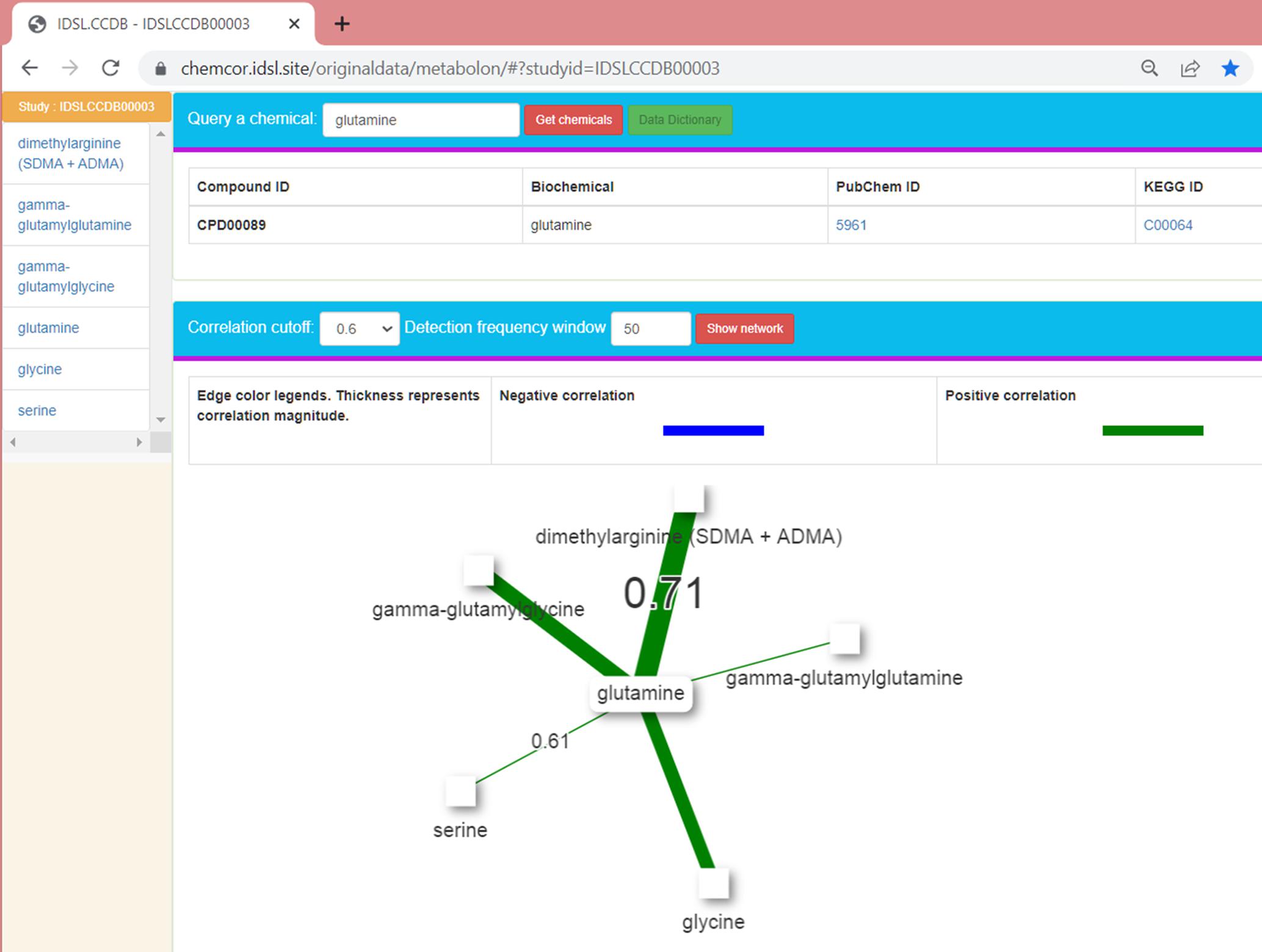

Supplement: FS3 [file NIHMS1811040-supplement-FS3.jpg]

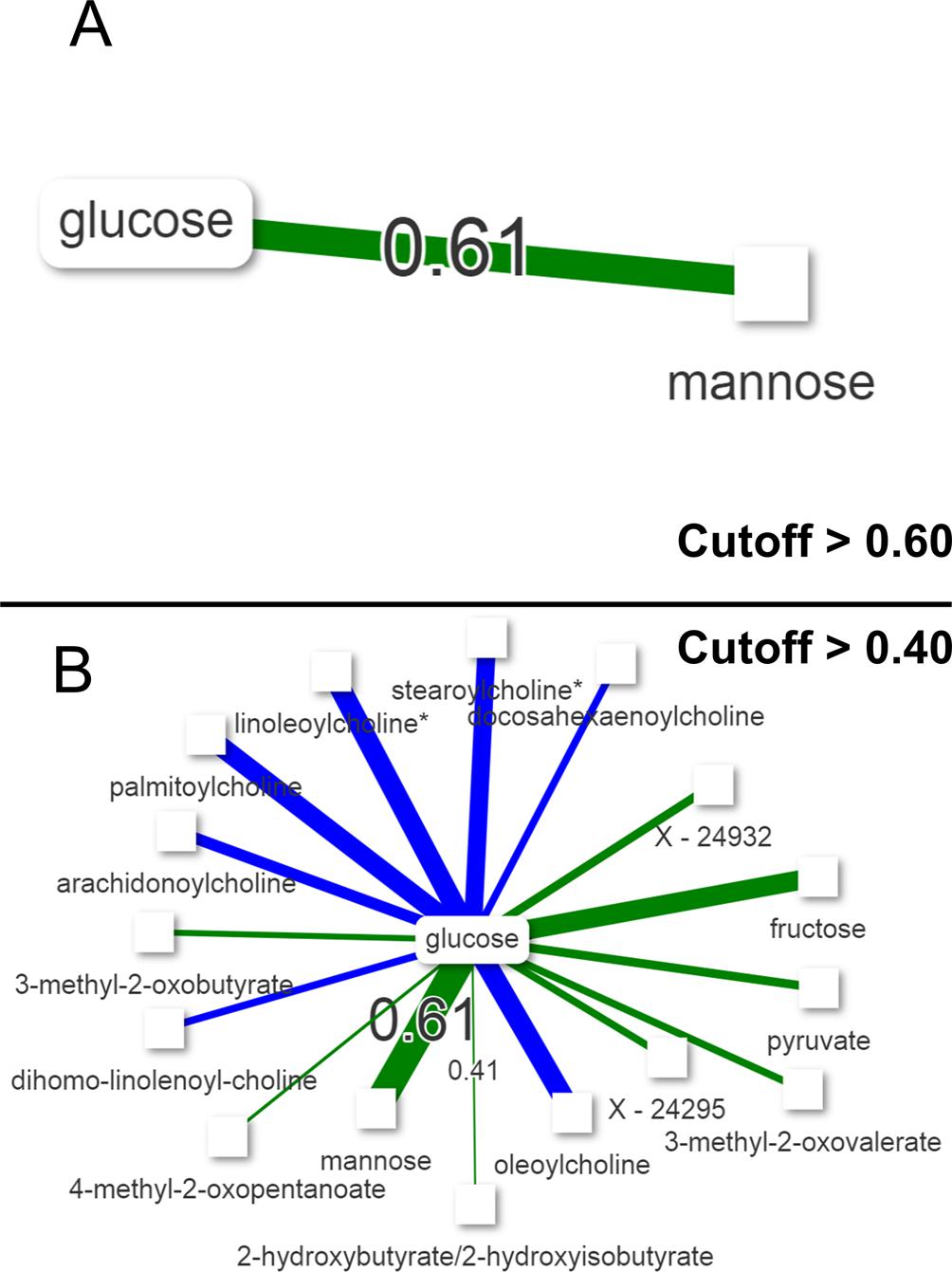

Supplement: FS2 [file NIHMS1811040-supplement-FS2.jpg]

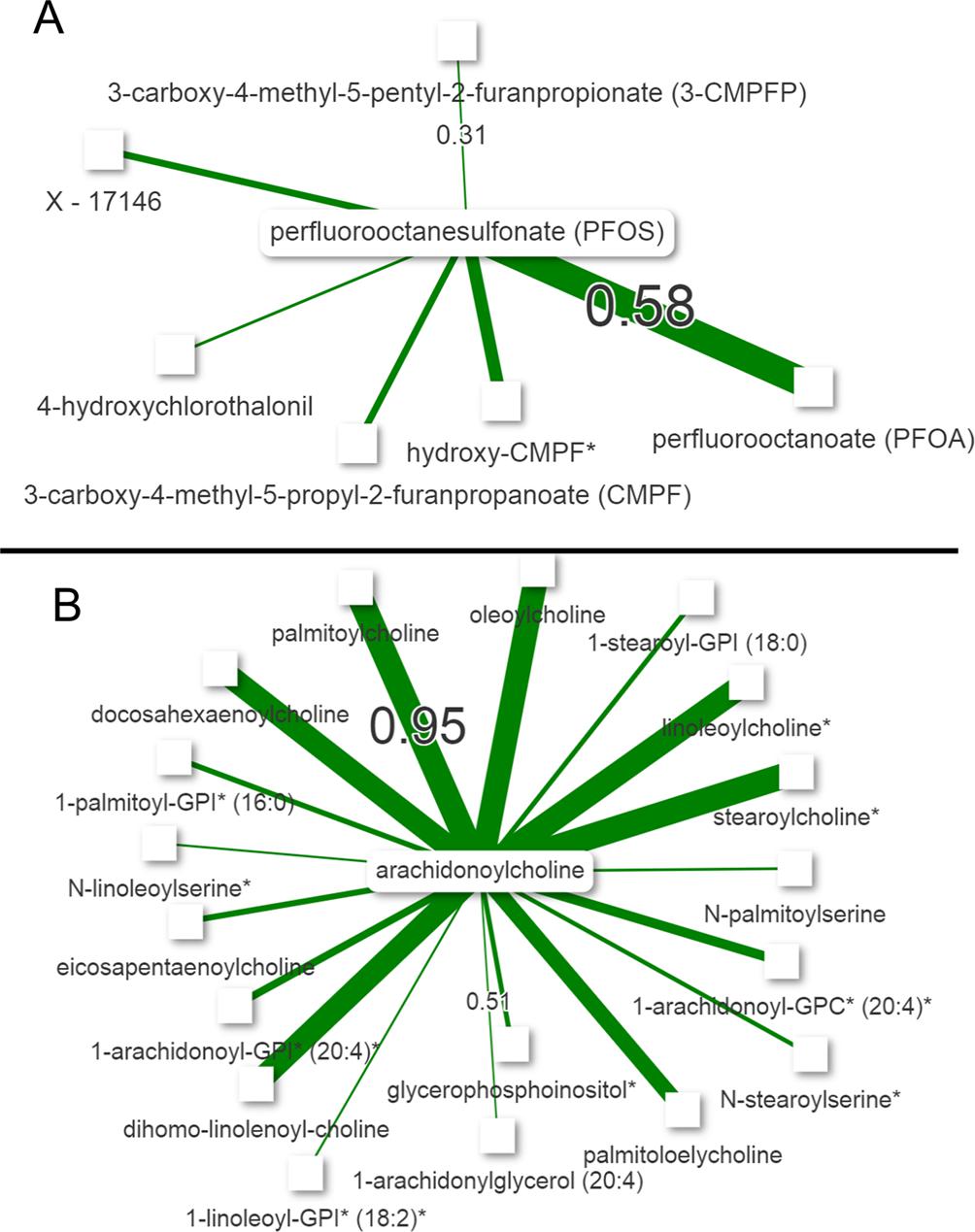

Supplement: FS1 [file NIHMS1811040-supplement-FS1.jpg]

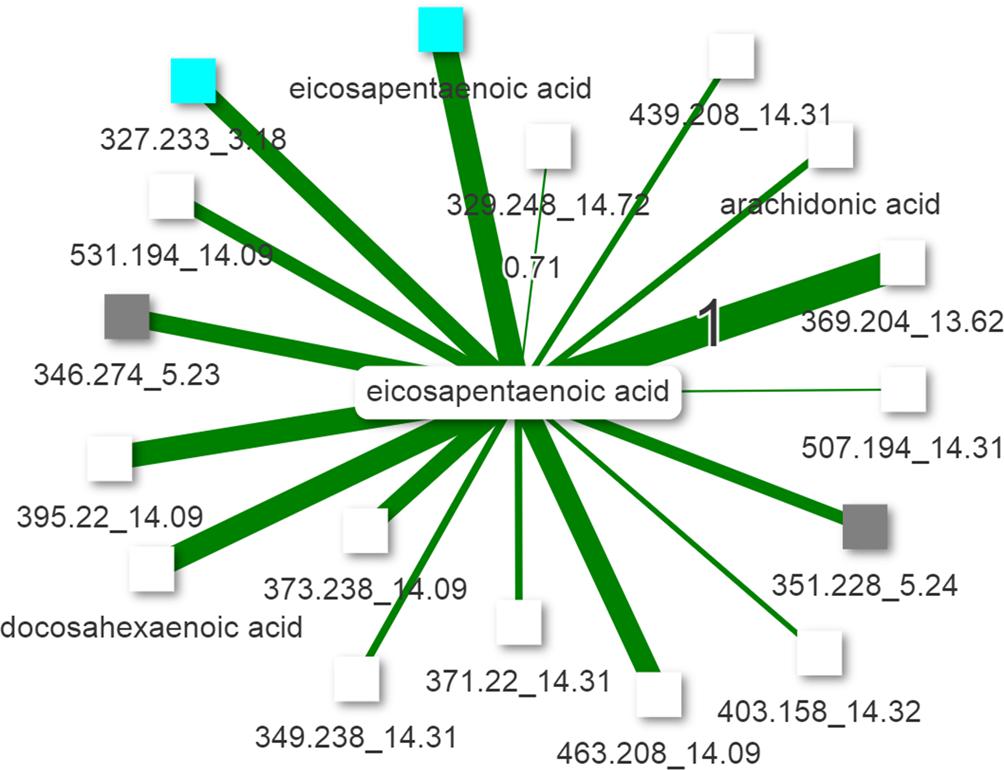

Supplement: FS6 [file NIHMS1811040-supplement-FS6.jpg]

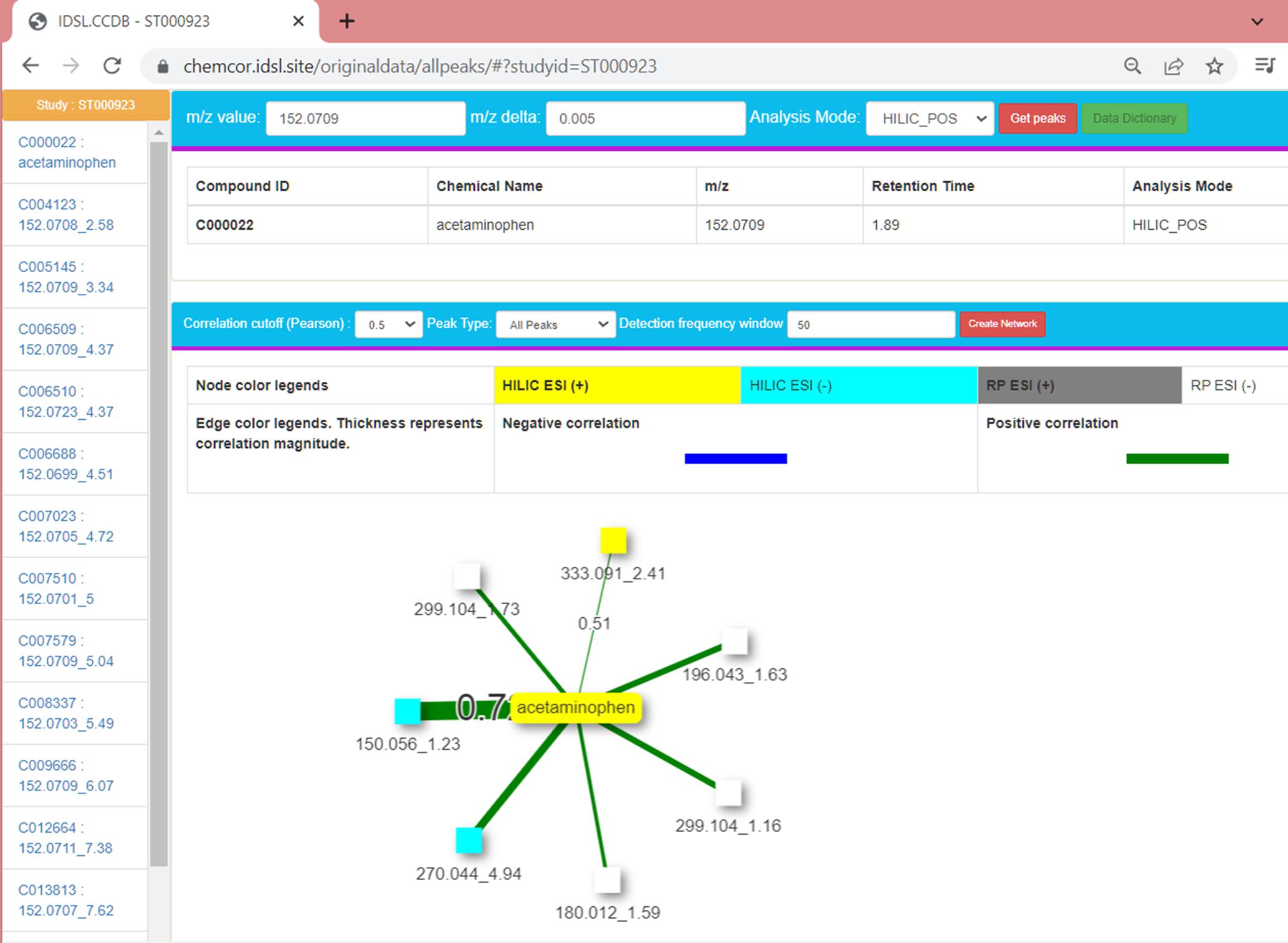

Supplement: FS4 [file NIHMS1811040-supplement-FS4.jpg]

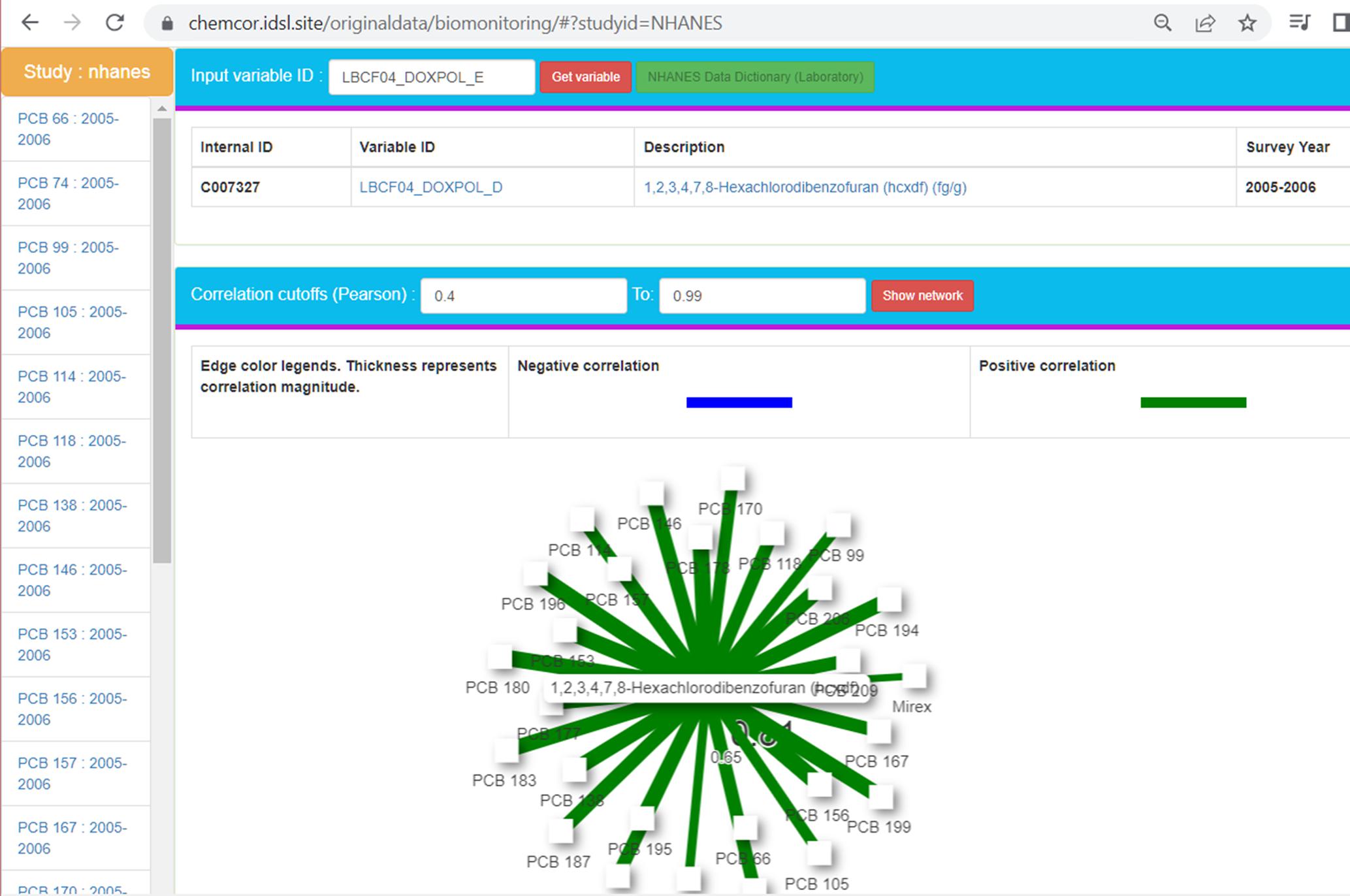

Supplement: FS5 [file NIHMS1811040-supplement-FS5.jpg]

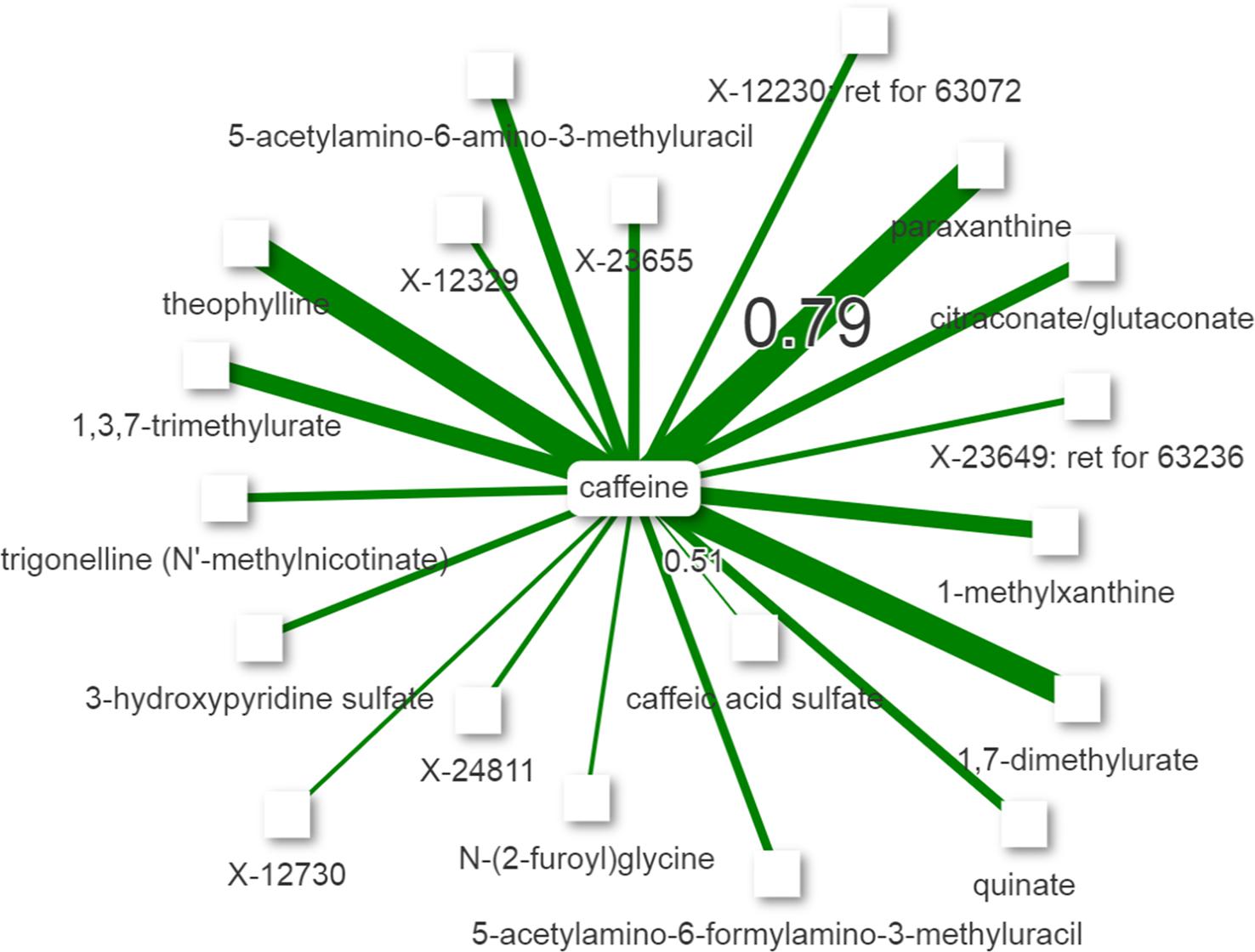

Supplement: FS7 [file NIHMS1811040-supplement-FS7.jpg]

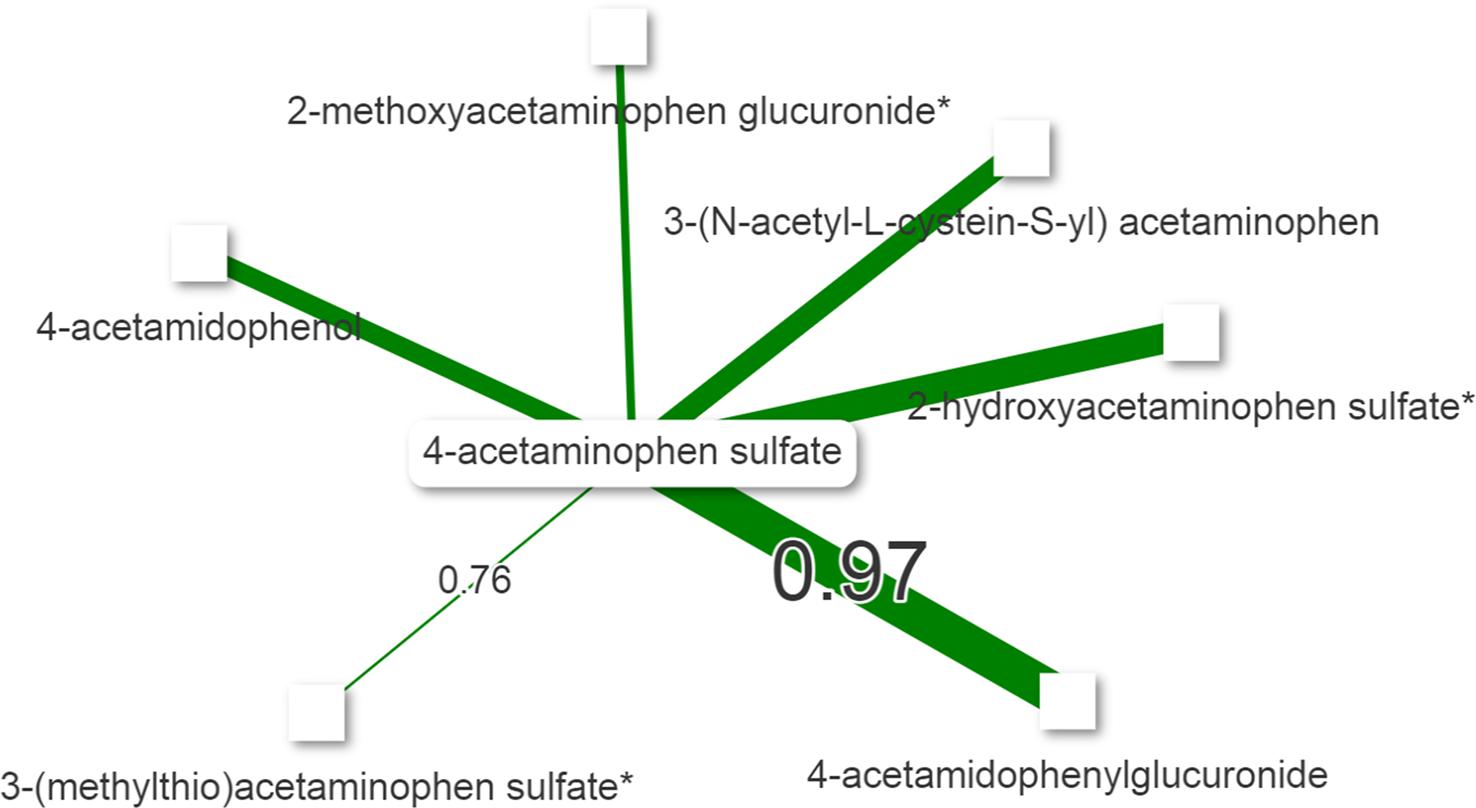

Supplement: FS8 [file NIHMS1811040-supplement-FS8.jpg]
